# Does Surgical Approach Influence Benchmark Attainment in Intrahepatic Cholangiocarcinoma?

**DOI:** 10.1002/wjs.70491

**Published:** 2026-07-24

**Authors:** Delphine Gastineau, Claire Goumard, Alexandre Doussot, Marc‐Antoine Allard, Raphael Venezia, Serena Langella, Guillaume Millet, Ruth Bustamante, Christian Hobeika, Rami Rhaiem, Stylianos Tzedakis, Heithem Jeddou, Bastien Le Floch, Alain Valverde, Nicolas Peru, Patrick Pessaux, Fabio Giannone, Jean Yves Mabrut, Kayvan Mohkam, Antonio Sa Cunha, Chady Salloum, Pierre Yves Blanc, Bertrand Le Roy, Takayuki Kawai, Olivier Farges, Stefan Hofmeyr, David Fuks, Stéphanie Truant, Daniel Cherqui, Alessandro Ferrero, Emmanuel Boleslawski, Jean‐Marc Regimbeau, Eric Vibert, Daniele Sommacale, Olivier Soubrane, Olivier Scatton, Alexis Laurent, Raffaele Brustia, AFC‐ICC‐2009, AFC‐LLR‐2018, PRS‐2019 and FRIES‐ACHBPT‐2024 Study group

**Affiliations:** ^1^ Department of Digestive and Hepato‐pancreatic‐biliary Surgery DMU CARE, AP‐HP Hôpitaux Universitaires Henri Mondor Créteil France; ^2^ Faculté de Santé GRCT OPTIMA Univ Paris Est Créteil Créteil France; ^3^ Department of Hepatobiliary and Liver Transplantation Surgery AP‐HP Hôpital Pitié Salpêtrière, CRSA Sorbonne Université Paris France; ^4^ Department of Digestive and Surgical Oncology Liver Transplantation Unit CHU Besancon Besançon France; ^5^ Hôpital Paul Brousse Centre Hépato‐Biliaire AP‐HP Villejuif France; ^6^ Inserm Université Paris‐Saclay UMRS 1193 FHU Hepatinov Villejuif France; ^7^ Department of General and Oncological Surgery Ospedale Mauriziano Torino Italy; ^8^ Department of Digestive Surgery and Transplantation Univ. Lille CHRU Lille Lille France; ^9^ Department of Hepatobiliary Surgery APHP University Hospitals Paris Nord Val de Seine Clichy France; ^10^ Department of Hepatobiliary, Pancreatic and Digestive Surgery Robert Debré University Hospital University Reims Champagne‐Ardenne Reims France; ^11^ Department of Hepatobiliary, Digestive and Endocrine Surgery Université Paris Cité Cochin Hospital, AP‐HP Paris France; ^12^ Department of Hepatobiliary and Digestive Surgery Pontchaillou University Hospital Rennes 1 University Rennes France; ^13^ Department of Digestive Surgery Groupe Hospitalier Diaconesses Croix Saint Simon Paris France; ^14^ Department of Digestive Surgery Strasbourg University Hospital Strasbourg France; ^15^ Department of Hepatobiliopancreatic Surgery and Liver Transplantation Hospital Croix Rousse Lyon France; ^16^ Department of Visceral and Digestive Surgery Centre Hospitalier Universitaire de Saint‐Etienne Saint‐Etienne France; ^17^ Department of Surgery Graduate School of Medicine Kyoto University Kyoto Japan; ^18^ Division of Surgery Faculty of Medicine and Health Sciences Stellenbosch University and Tygerberg Academic Hospital Cape Town South Africa; ^19^ SSPC—Clinical Research Unit University of Picardie Jules Verne Department of Digestive Surgery Amiens University Medical Center Amiens France; ^20^ Department of Digestive, Oncologic and Metabolic Surgery Institut Mutualiste Montsouris Université Paris Descartes Paris France

**Keywords:** intra‐hepatic cholangiocarcinoma, liver surgery, long‐term outcomes, lymphadenectomy, node dissection

## Abstract

**Background:**

Intrahepatic cholangiocarcinoma (ICC) is a rare malignancy with rising incidence and poor prognosis. Surgical resection remains the only curative option, with outcomes driven by R0 margins and lymphadenectomy (LND). While open liver resection (OLR) is standard, laparoscopic (LLR) and robotic liver resection (RLR) are increasingly used, with concerns about their oncologic adequacy.

**Objective:**

To assess the association between composite surgical benchmark attainment (R0 + LND) and short‐ and long‐term outcomes after ICC resection, stratified per surgical approach.

**Methods:**

A retrospective multicenter analysis was conducted including 240 patients treated from 2010 to 2024. The primary endpoint was achievement of both R0 resection and LND. Secondary endpoints included 90‐day mortality, overall (OS), and disease‐free survival (DFS).

**Results:**

Among the 240 included patients 38.8% met surgical benchmarks (R0 + LND), with rates varying by approach: OLR 51.1%, RLR 44.4%, and LLR 14.5% (*p* < 0.001). 90‐day mortality was 5.0% (12/240) overall, with no significant difference between surgical approaches (*p* = 0.219). One and 3‐year overall survival was 79%/30% for OLR, 95%/89% for LLR, and 90%/81% for RLR (*p* < 0.001). Multivariable analysis identified tumor multiplicity as an independent predictor of mortality (HR = 2.18, *p* = 0.003), while LLR was independently protective (HR = 0.15, *p* < 0.001); RLR showed a non‐significant protective trend.

**Conclusion:**

Although OLR and RLR more frequently met oncological benchmark criteria, only LLR was independently associated with improved overall survival, highlighting a potential disconnect between benchmark adherence and long‐term outcomes in ICC.

## Graphical abstract

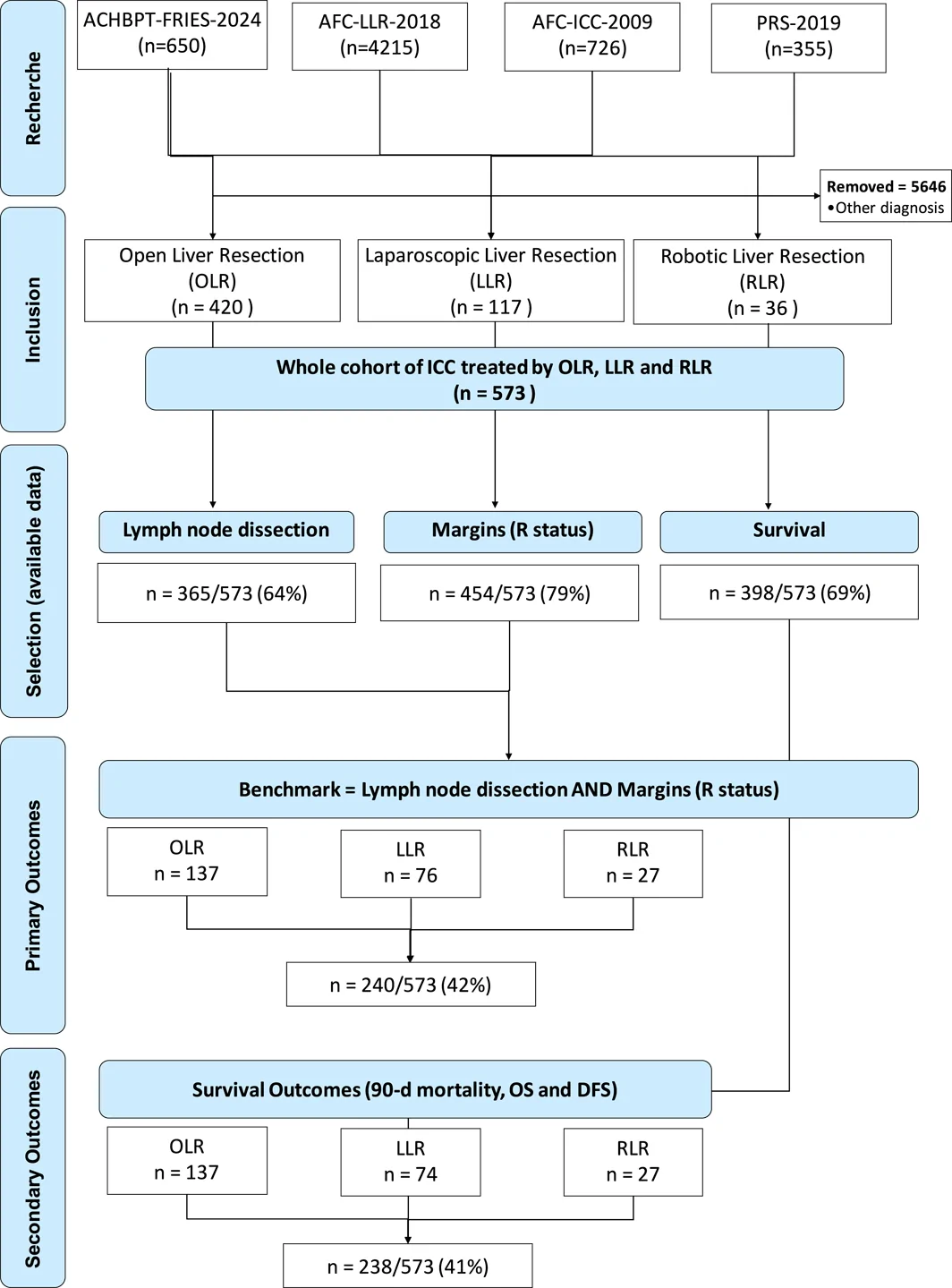

## 1 Introduction

Intrahepatic cholangiocarcinoma (ICC) is a rare malignancy characterized by a poor prognosis [1, 2], with its incidence increasing in Western countries [3, 4]. For patients eligible for surgical resection, long‐term outcomes are primarily determined by negative surgical margins (R0) and lymph node dissection (LND), which defines N status [5, 6], together with adverse biological markers, high tumor burden, and aggressive histopathological features [7]. There is considerable heterogeneity in threshold definitions for both variables, with no consensus on the minimum standard for resection margins [5, 8, 9] or the number of lymph nodes required [6, 9, 10]. Recent benchmark data for ICC report an R1 resection rate of ≤ 27.5% and LND performed in only 42.8% of patients, underscoring its limited adoption in clinical practice [6].

While open liver resection (OLR) remains the gold standard [11], laparoscopic liver resection (LLR) has gained increasing acceptance in terms of oncological efficacy [12, 13], except for LND [14]. The emergence of robotic liver resection (RLR) offers the potential to minimize this gap, by enabling more precise LND and optimizing margin status [15], enhancing oncologic outcomes [16].

This study aims to evaluate whether surgical benchmark attainment—assessed using a composite outcome defined by the combined achievement of R0 resection and LND—was associated with short‐ and long‐term outcomes after ICC resection, with analyses stratified by surgical approach to reflect real‐world practice.

## 2 Methods

A retrospective, comparative effectiveness study design was set to investigate oncological outcomes across three different surgical techniques (OLR, LLR, RLR) within the target population of patients affected by ICC.

### 2.1 Settings and Data Source

This study was designed in June 2024 in accordance with the *Strengthening the Reporting of Observational Studies in Epidemiology (STROBE) Statement* and guidelines [17].

Given the limited experience of individual centers, data from four independent multicenter databases were merged.

1. The FRIES‐ACHBPT‐2024 study group was created in 2023 under the auspices of the *Association Française de Chirurgie Hépato‐Bilio‐Pancréatique et Transplantation* (ACHBPT) to produce a retrospective French registry of RLR (French Robotic lIvEr Surgery) ACHBT‐L001. Data on 650 consecutive patients undergoing RLR from 2010 to 2024 were retrospectively collected from 10 HPB French centers [18]. The nested cohort of patients treated for ICC was selected for this study.
2. The AFC‐LLR‐2018 study group was created in 2018 under the auspices of the *Association Française de Chirurgie* (AFC) to produce a retrospective registry of LLR. Data on 4215 consecutive patients undergoing LLR from 1995 to 2018 were retrospectively collected from 29 European institutions. The nested cohort of patients treated for ICC was selected for this study.
3. The Preoperative Risk Score (PRS) study group was organized to externally validate a published score for prediction of long‐term outcomes after surgery for ICC. Eleven international HPB centers provided data on 355 patients treated by OLR or LLR from 2001 to 2018 for ICC [19].
4. The AFC‐ICC‐2009 study group was created in 2009 under the auspices of the AFC to produce a registry of patients operated for ICC. Data on all consecutive patients undergoing OLR with curative intent for ICC from 1989 to 2009 were collected from a dedicated multi‐institutional database from 24 university hospitals, as reported elsewhere [8].

**FIGURE 1:**
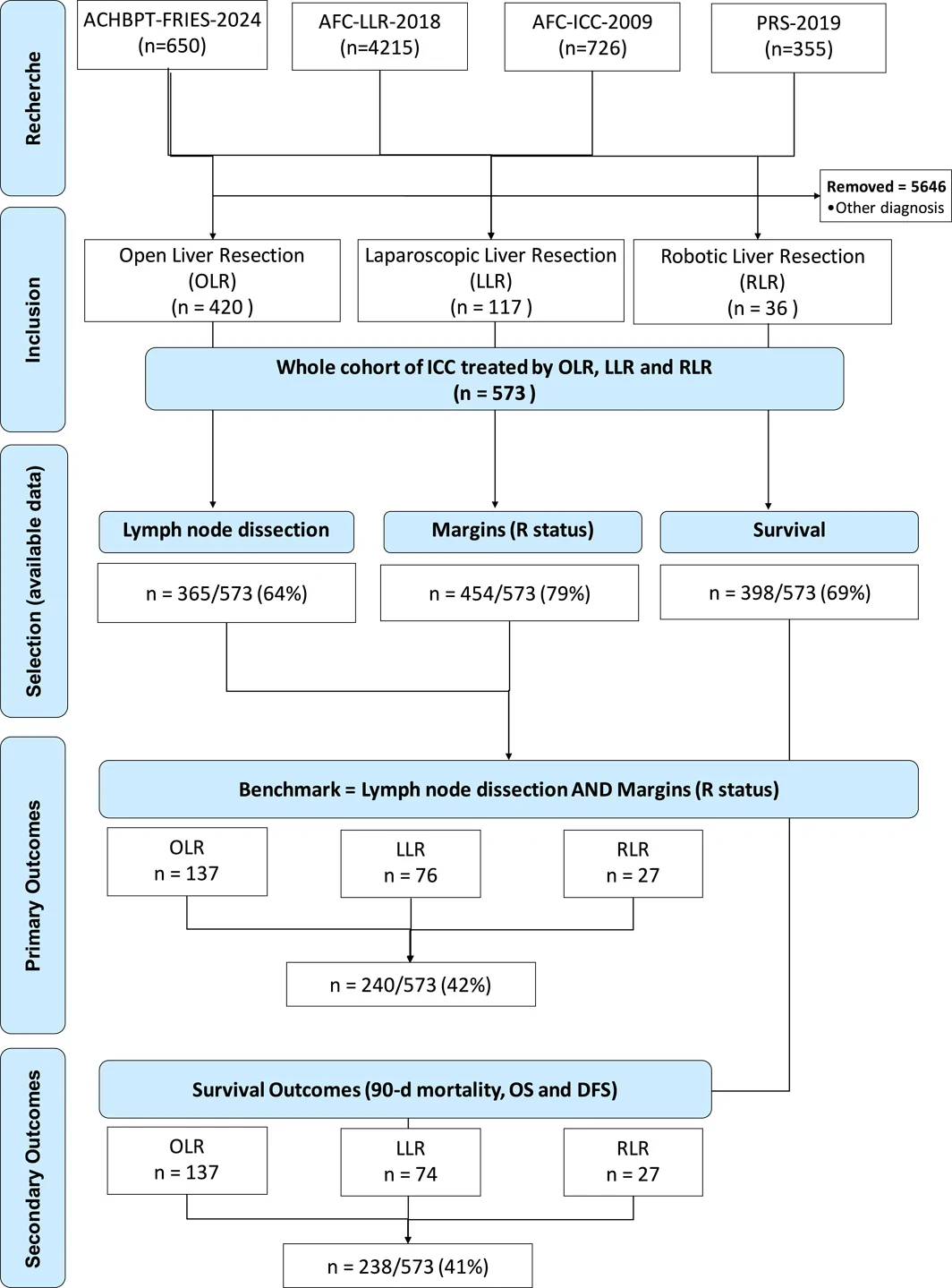
Flowchart of included patients.

Patients treated by OLR, LLR or RLR from January 2010 up to August 2024 were included, with a minimum follow‐up period of 6 months (Figure 1, flowchart).

Patients from each database were first screened for duplicates and data completeness before being merged into a raw working dataset. They were then further assessed based on the following inclusion criteria:

— Underwent OLR, LLR or RLR with curative intent (R0 or R1) via hepatectomy, with or without an associated procedure.
— Had a diagnosis of ICC, excluding hepatocholangiocarcinomas, cystadenocarcinomas, gallbladder cancer, and hilar cholangiocarcinomas.

### 2.2 Study Outcomes

#### 2.2.1 Primary Outcome: Composite Oncological Benchmark Attainment

The primary endpoint was a composite measure of optimal oncological surgery, defined as the simultaneous achievement of both:

◦ Negative surgical margins (R0)
◦ Lymph node dissection (LND)

Patients fulfilling *both* components were classified as having achieved the benchmark. This composite outcome was chosen to provide a holistic assessment of surgical quality, integrating two cornerstone principles of ICC resection. Data completeness allowed primary outcome assessment in 240 patients.

#### 2.2.2 Secondary Survival Outcomes

◦ Mortality at 90‐day postoperative.
◦ Overall survival (OS) and disease‐free survival (DFS) at 1 and 3 years.

Data completeness allowed survival outcome assessment in 238 patients.

### 2.3 Variables of Interest and Data Managment

#### 2.3.1 Surgical Variables

The extent of LS was classified according to the Institut Mutualiste Montsouris (IMM) Difficulty classification [20, 21], with three increasing levels of difficulty according to the number, location and difficulty of resected liver segments.

#### 2.3.2 Oncological Outcomes

Resection margin status was determined locally based on histopathological examination of the surgical specimen, performed by institutional hepatobiliary pathologists according to national standards. Multiple margin sections were examined per specimen, and R0 (no microscopic tumor at the inked margin) and R1 (microscopic involvement) were defined using uniform criteria across centers. Given the 15‐year retrospective multicenter design, centralized pathological review was not feasible, and validated institutional reports were accepted as recorded. Patients were stratified into R0 and R1 groups for survival and recurrence analyses; those with macroscopic residual disease (R2) were excluded. The reported benchmark [6] for ICC include a positive resection margin rate of ≤ 27.5%, with no consensus on the minimum standard of margins [5].

LND is typically defined as the removal of regional lymph nodes (station 8, 12 and 13). Standardized staging guidelines for ICC with LND were first introduced in the AJCC 7th edition (2010) and later refined in the 8th edition (2017), which recommended evaluating a minimum of four—and more recently six—lymph nodes for adequate staging. However, recent benchmark data reported that LND was performed in only 42.8% of patients and included ≥ 3 lymph nodes. This historical variability in node‐count thresholds explains our pragmatic choice of a “LND performed: yes/no” criterion rather than a specific node‐count threshold for this multicenter study spanning 14 years. Given the heterogeneity of available databases and missing information in our study, lymph node dissection (LND) was considered to have been performed if at least one lymph node was examined and reported in the final pathology report, irrespective of nodal status (N0 or N+). Patients with no lymph nodes described in the pathology report were classified as “no LND”.

#### 2.3.3 Postoperative Variables

Due to the nature of the data source, which includes information from over 40 centers, investigators did not have access to individual patient data, including postoperative complications or length of stay. The only hard‐outcome assessed was 90‐day postoperative mortality, which has been recently benchmarked at ≤ 4.8% [6].

#### 2.3.4 Long Term Oncological Outcomes

Although follow‐up schedules were center‐specific, all participating institutions adhered to national recommendations for postoperative surveillance of ICC, which typically include cross‐sectional imaging every 3–6 months during the first 2 years and every 6–12 months thereafter, with CA 19‐9 assessments when clinically indicated. Recurrence was defined as the detection of new tumor lesions after curative‐intent surgical resection, confirmed by radiological or histopathological evaluation. Survival analyses were based on the dates of imaging‐confirmed events of recurrence. Recurrence was classified as local (intrahepatic recurrence within the liver remnant), regional (involvement of regional lymph nodes), or distant (extrahepatic metastasis to organs such as the peritoneum, lungs, or bones). Tumor recurrence was assessed using contrast‐enhanced CT and/or MRI every 3–6 months during follow‐up, with additional imaging modalities (PET‐CT, ultrasound) and CA 19‐9 biomarker evaluation when clinically indicated. Disease free survival (DFS) was defined as the interval from the date of surgery to the date of first confirmed recurrence, with patients without recurrence censored at last follow‐up.

#### 2.3.5 Data Management

Perioperative variables from each database were consolidated into a single dataset for analysis. Each patient was anonymized and assigned a unique alphanumeric identifier. The dataset was stored on a secure, password‐protected computer with restricted access. Data management adhered to the MR004 reference methodology for personal data processing and protection, as required by the French data protection authority (*Commission Nationale de l'Informatique et des Libertés*).

### 2.4 Statistical Analysis Methods

#### 2.4.1 Statistical Software

Data managing, statistical evaluation and analysis were performed with R software (version 4.4.2 or higher, the R Foundation for Statistical Computing. www.cran.r‐project.org, Vienna, Austria). All the packages, libraries and functions refer to the R Software.

#### 2.4.2 Descriptive Statistics

Categorical variables were reported as percentages, while continuous variables were summarized as means and standard deviation (SD) or median and range for discrete variables, as appropriate. The Student's *t‐*test or Mann‐Whitney *U* test were used for comparisons of quantitative variables as appropriate, whereas a *χ* ^2^ test or Fisher's exact test was used to compare categorical data.

#### 2.4.3 Survival Analyses

Kaplan–Meier curves for OS and DFS before and after matching were created through survfit and ggsurvplot functions from survminer and survival packages, with three strata corresponding to OLR, LLR and RLR groups.

#### 2.4.4 Definition of Variables Predicting Death Within the Whole Cohort

Unadjusted hazard ratios (HRs) and 95% confidence intervals (95% CI) were calculated by Cox proportional hazards regression analysis for peri‐operative variables associated with death or recurrence. Variables with a *p* value < 0.1 (as well as those considered clinically relevant) were entered in a multivariate Cox model to identify factors independently associated with death or recurrence. The final model expressed the adjusted HRs and 95%CI. A Leave‐One‐Center‐Out (LOCO) sensitivity analysis was performed by sequentially excluding each center and refitting the multivariable Cox model to assess the robustness of the association between surgical approach and overall survival.

## 3 Results

### 3.1 Participants

A total of 240 patients from 42 participating centers met the inclusion criteria and constituted the study cohort, including 137 (57.1%) open liver resections (OLR), 76 (31.7%) laparoscopic liver resections (LLR), and 27 (11.3%) robotic liver resections (RLR) (Figure 1). The distribution of sex was comparable across groups (50.8% female overall; *p* = 0.219). The mean age was 63.2 ± 12.0 years, with no significant difference between surgical approaches (*p* = 0.531). Similarly, BMI was comparable between groups (mean 26.8 ± 4.9 kg/m^2^; *p* = 0.194). However, obesity (defined as BMI > 30 kg/m^2^) was significantly more frequent in the LLR (26.3%) and RLR (29.6%) groups compared to OLR (10.2%) (*p* < 0.001). The study population was followed for a median duration of 16.1 months (IQR: 6.99–30.3 months).

Liver‐related comorbidities differed markedly between approaches: the prevalence of cirrhosis was higher in the LLR (29.6%) and RLR (34.5%) groups compared to OLR (1.9%) (*p* < 0.001). Likewise, metabolic syndrome was significantly more frequent in patients undergoing LLR (32.9%) and RLR (40.7%) compared to OLR (10.9%) (*p* < 0.001). ASA physical status classification was similar across the three groups (*p* = 0.514).

Tumor characteristics varied by approach. Patients undergoing minimally invasive surgery (LLR and RLR) were more likely to have a single lesion (89.5% and 88.9%, respectively) compared to OLR (70.1%) (*p* = 0.002), and tumors were significantly smaller in the LLR (4.64 ± 2.76 cm) and RLR (5.31 ± 2.72 cm) groups than in the OLR group (7.34 ± 3.56 cm) (*p* < 0.001). Consistently, the proportion of tumors larger than 5 cm was higher in the OLR group (68.6%) compared to LLR (30.3%) and RLR (37.0%) (*p* < 0.001).

The complexity of procedures also varied significantly according to the IMM surgical difficulty classification (*p* < 0.001), with IMM class II–III resections representing 96.3% of RLR, 67.1% of LLR, and 87.6% of OLR. Blood loss was similar across groups (*p* = 0.803), and transfusion rates did not differ significantly (*p* = 0.238).

**FIGURE 2:**
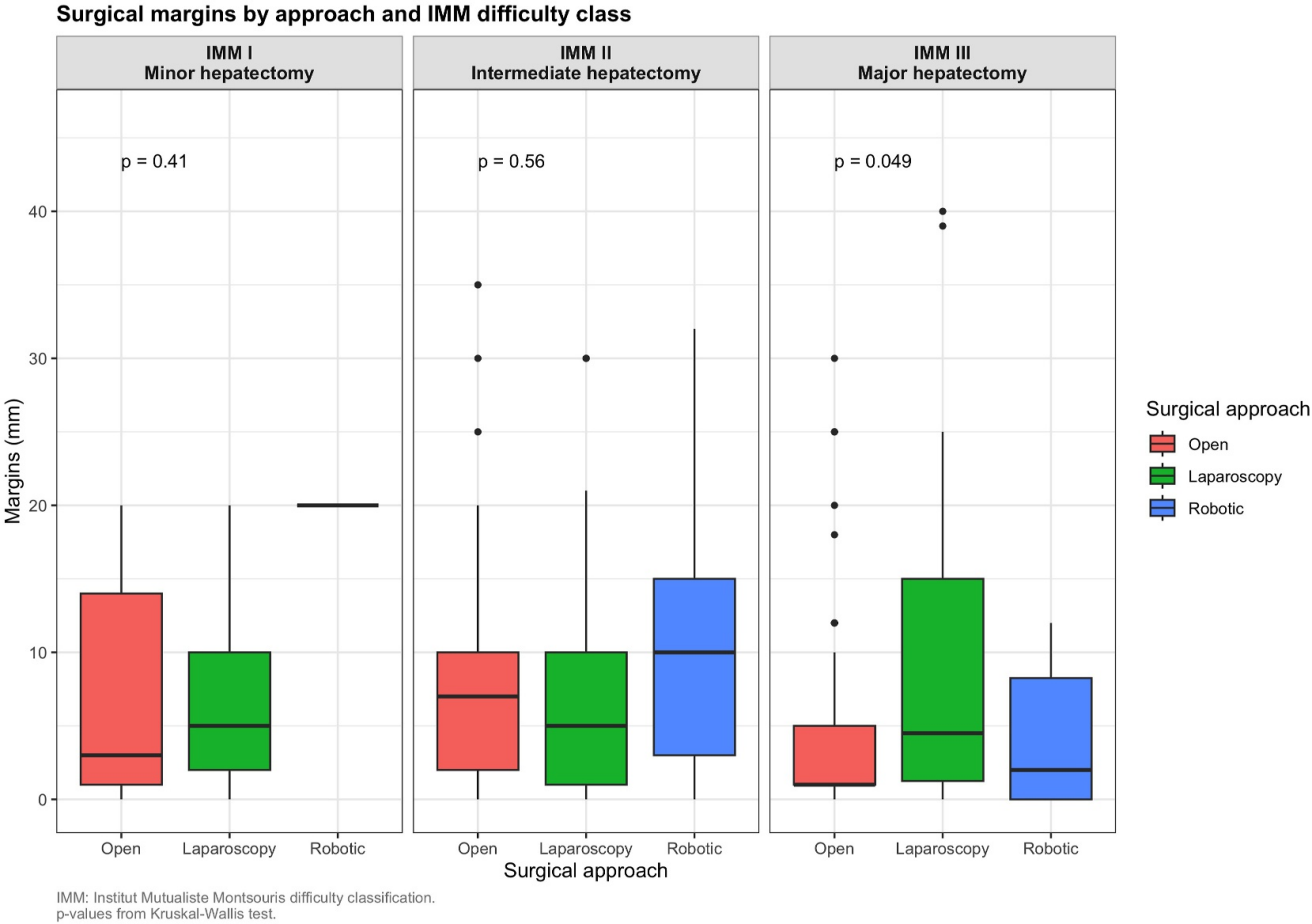
Median margins in millimeters, according to the surgical approach and difficulty.

Lymphadenectomy (LND) was performed in 167 patients (69.6%), with a significantly higher rate in the OLR group (97.8%) compared to RLR (59.3%) and LLR (22.4%) (*p* < 0.001). R0 resection was achieved in 60.4% of patients, with the highest rates in the LLR (72.4%) and RLR (66.7%) groups, compared to OLR (52.6%) (*p* = 0.014). Resection margins were significantly wider in the LLR (9.56 ± 12.1 mm) and RLR (9.30 ± 11.3 mm) groups than in the OLR group (5.43 ± 7.7 mm) (*p* = 0.007). The composite benchmark of oncological quality—defined as R0 resection with LND—was achieved in 38.8% of the cohort overall, with significant differences between groups: 51.1% in OLR, 14.5% in LLR, and 44.4% in RLR (*p* < 0.001). Further details are provided in Table 1. Moreover, no differences in median margins were observed after stratifying per surgical difficulty (Figure 2).

**TABLE 1:** Preoperative, intraoperative and postoperative characteristics of the study cohort.

Variable	Overall (*n* = 240)	Open (*n* = 137)	Laparoscopy (*n* = 76)	Robotic (*n* = 27)	*p*‐value
Preoperative characteristics					
Age, years (mean ± SD)	63.19 (11.98)	62.68 (11.32)	63.27 (13.29)	65.52 (11.64)	0.531
Sex, *n* (%)					0.219
Female	122 (50.8)	75 (54.7)	37 (48.7)	10 (37.0)	
Male	118 (49.2)	62 (45.3)	39 (51.3)	17 (63.0)	
BMI, kg/m^2^ (mean ± SD)	26.75 (4.93)	26.08 (4.40)	27.03 (5.16)	27.95 (5.63)	0.194
Obesity (BMI > 30), *n* (%)	42 (17.5)	14 (10.2)	20 (26.3)	8 (29.6)	< 0.001
Cirrhosis, *n* (%)	37 (15.4)	3 (2.2)	24 (31.6)	10 (37.0)	< 0.001
Metabolic syndrome, *n* (%)	51 (21.2)	15 (10.9)	25 (32.9)	11 (40.7)	< 0.001
ASA class, *n* (%)					0.514
I	33 (13.8)	23 (16.8)	6 (7.9)	4 (14.8)	
II	116 (48.3)	67 (48.9)	36 (47.4)	13 (48.1)	
III	90 (37.5)	46 (33.6)	34 (44.7)	10 (37.0)	
IV	1 (0.4)	1 (0.7)	0 (0.0)	0 (0.0)	
Tumor characteristics					
Single lesion, *n* (%)	188 (78.3)	96 (70.1)	68 (89.5)	24 (88.9)	0.002
Largest tumor size, cm (mean ± SD)	6.27 (3.47)	7.34 (3.56)	4.64 (2.76)	5.31 (2.72)	< 0.001
Tumor > 5 cm, *n* (%)	127 (52.9)	94 (68.6)	23 (30.3)	10 (37.0)	< 0.001
Intraoperative characteristics					
IMM surgical difficulty, *n* (%)					< 0.001
I (minor)	34 (14.2)	11 (8.0)	22 (28.9)	1 (3.7)	
II (intermediate)	62 (25.8)	19 (13.9)	29 (38.2)	14 (51.9)	
III (major)	136 (56.7)	101 (73.7)	23 (30.3)	12 (44.4)	
Lymphadenectomy (LND), *n* (%)	167 (69.6)	134 (97.8)	17 (22.4)	16 (59.3)	< 0.001
Blood loss, mL (mean ± SD)	458 (513)	452 (385)	441 (569)	532 (497)	0.803
Transfusion, *n* (%)	58 (24.2)	36 (26.3)	19 (25.0)	3 (11.1)	0.238
Postoperative outcomes					
Resection margin, *n* (%)					0.014
R0 (negative)	145 (60.4)	72 (52.6)	55 (72.4)	18 (66.7)	
R1 (positive)	95 (39.6)	65 (47.4)	21 (27.6)	9 (33.3)	
Margin width, mm (mean ± SD)	7.17 (9.90)	5.43 (7.72)	9.56 (12.12)	9.30 (11.32)	0.007
Benchmark (R0 + LND), *n* (%)	93 (38.8)	70 (51.1)	11 (14.5)	12 (44.4)	< 0.001
90‐day postoperative mortality, *n* (%)	12 (5.0%)	10 (7.3%)	2 (2.6%)	0 (0%)	0.219^a^
Recurrence, *n* (%)	103 (42.9)	70 (51.1)	23 (30.3)	10 (37.0)	0.034
Disease‐free survival, months (mean ± SD)	15.59 (18.29)	15.20 (18.15)	17.20 (19.91)	13.02 (13.93)	0.554
Overall survival, months (mean ± SD)	20.01 (18.71)	20.64 (18.33)	19.39 (20.48)	18.59 (15.60)	0.823

*Note:* Age, BMI, blood loss, margin width, disease‐free and overall survival are expressed as mean ± standard deviation (SD). All other variables are reported as number (%).

Abbreviations: ASA, American Society of Anesthesiologists; BMI, Body Mass Index; IMM, Institut Mutualiste Montsouris Difficulty Classification; LND, Lymphadenectomy; R0, negative resection margin; R1, positive resection margin.

^a^ Fisher's exact test used given small cell counts.

### 3.2 Primary Outcome: Benchmark of Oncological Quality

**FIGURE 3:**
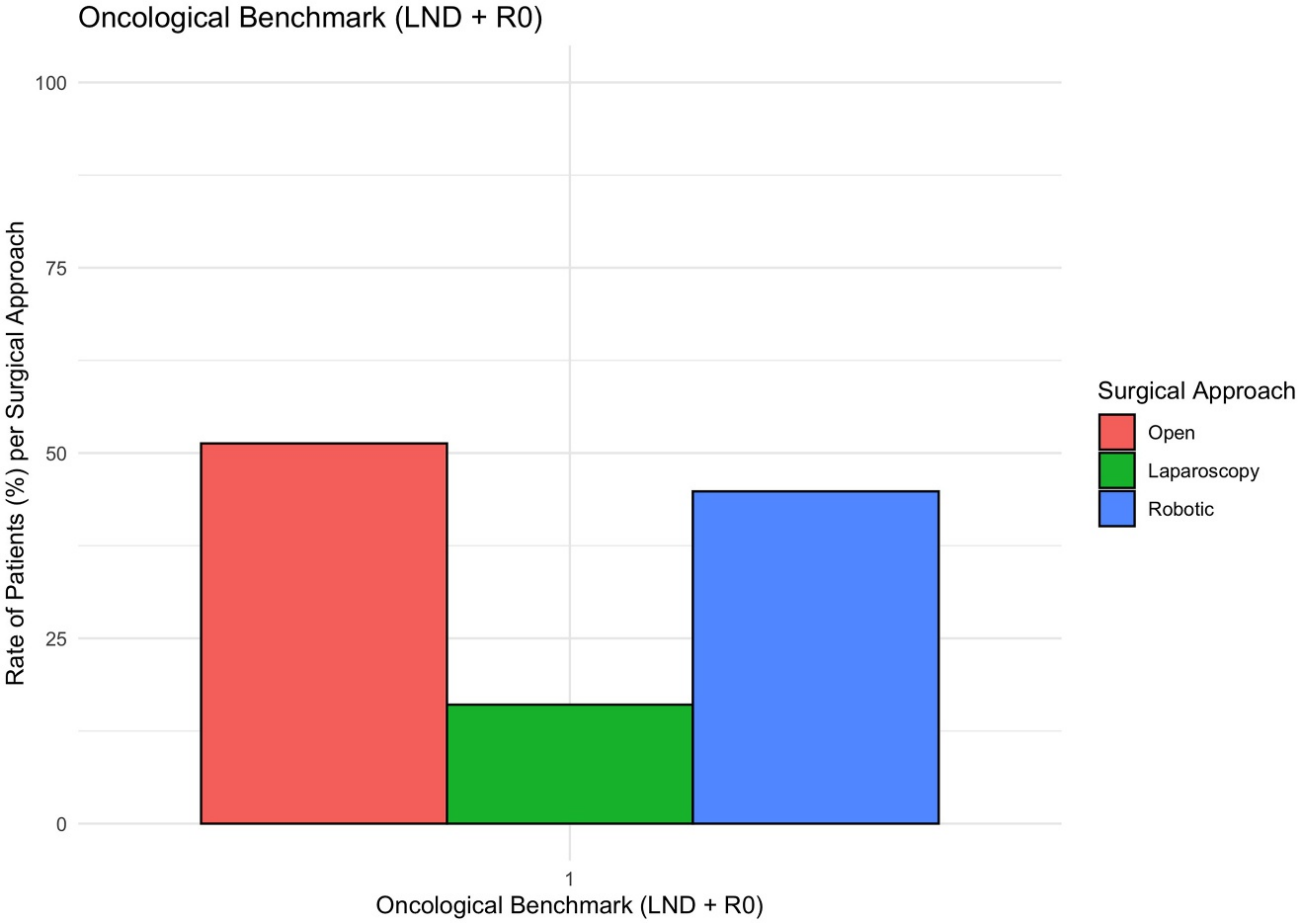
Rates of patients meeting the benchmark criteria (R0 and lymphadenectomy) according to the surgical approach: Open liver resection group (70/137, 51.1%), Laparoscopic Liver Resection group (11/76, 14.5%) and Robotic Liver Resection group (12/27, 44.4%) (*p* < 0.001).

Overall, 93 patients (38.8%) met the predefined benchmark criteria, with significant variation across surgical approaches (*p* < 0.001). The highest proportion was observed in the OLR group (70/137, 51.1%), followed by the RLR group (12/27, 44.4%) and the LLR group (11/76, 14.5%) (Figure 3).

### 3.3 Secondary Survival Outcomes

#### 3.3.1 90‐Day Postoperative Mortality

Ninety‐day postoperative mortality occurred in 12 patients (5.0%) across the entire cohort. Mortality rates were 7.3% in the OLR group (10/137), 2.6% in the LLR group (2/76), and 0% in the RLR group (0/27). No statistically significant difference was observed between surgical approaches (Fisher's exact test, *p* = 0.219). Causes of death were heterogeneous and predominantly related to severe sepsis or major cardiopulmonary events, with only two deaths attributable to early tumor recurrence within the first 90 days.

#### 3.3.2 Overall and Disease Free Survival (1 and 3 years)

**FIGURE 4:**
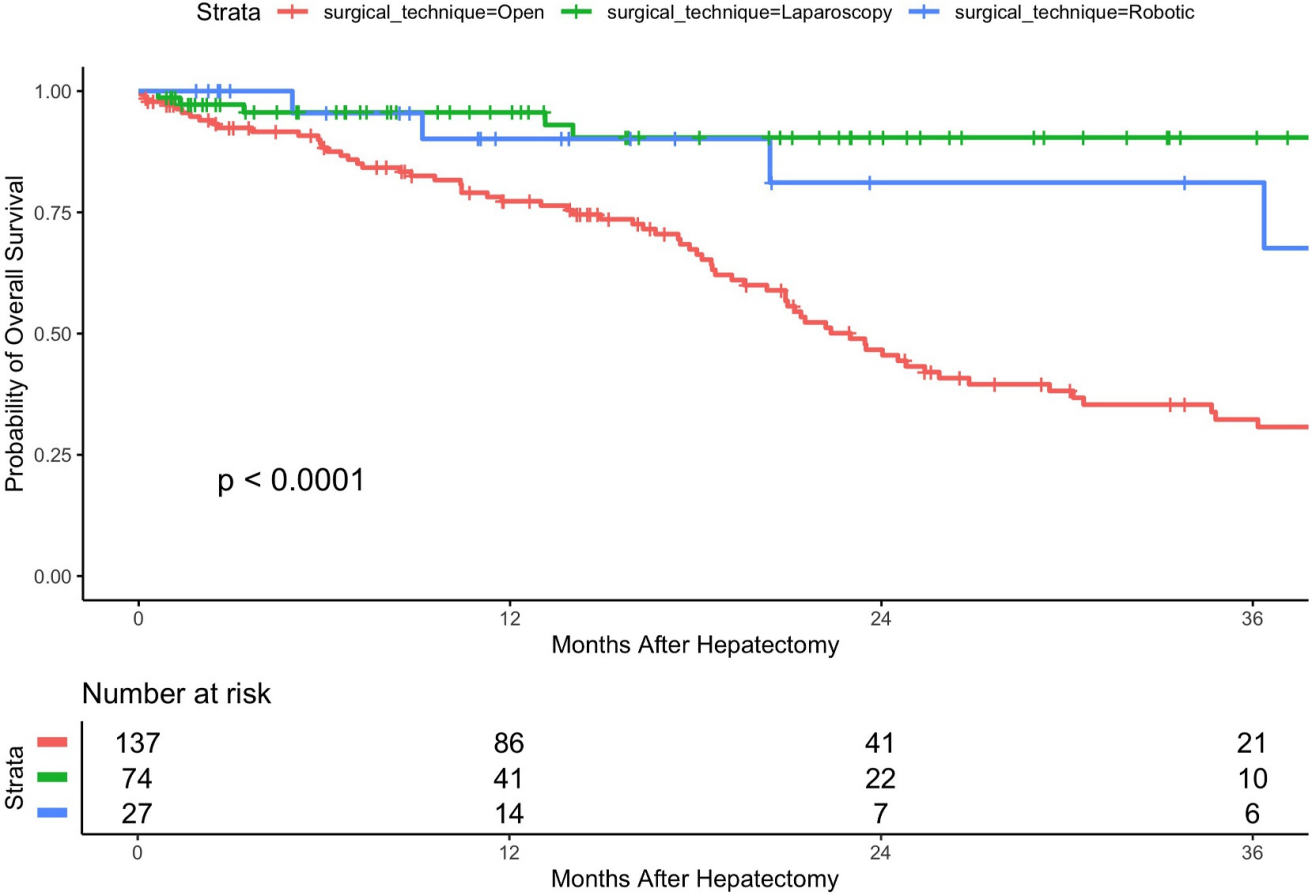
Kaplan‐Meier overall survival estimation at 1 and 3 years, according to the surgical approach.

**FIGURE 5:**
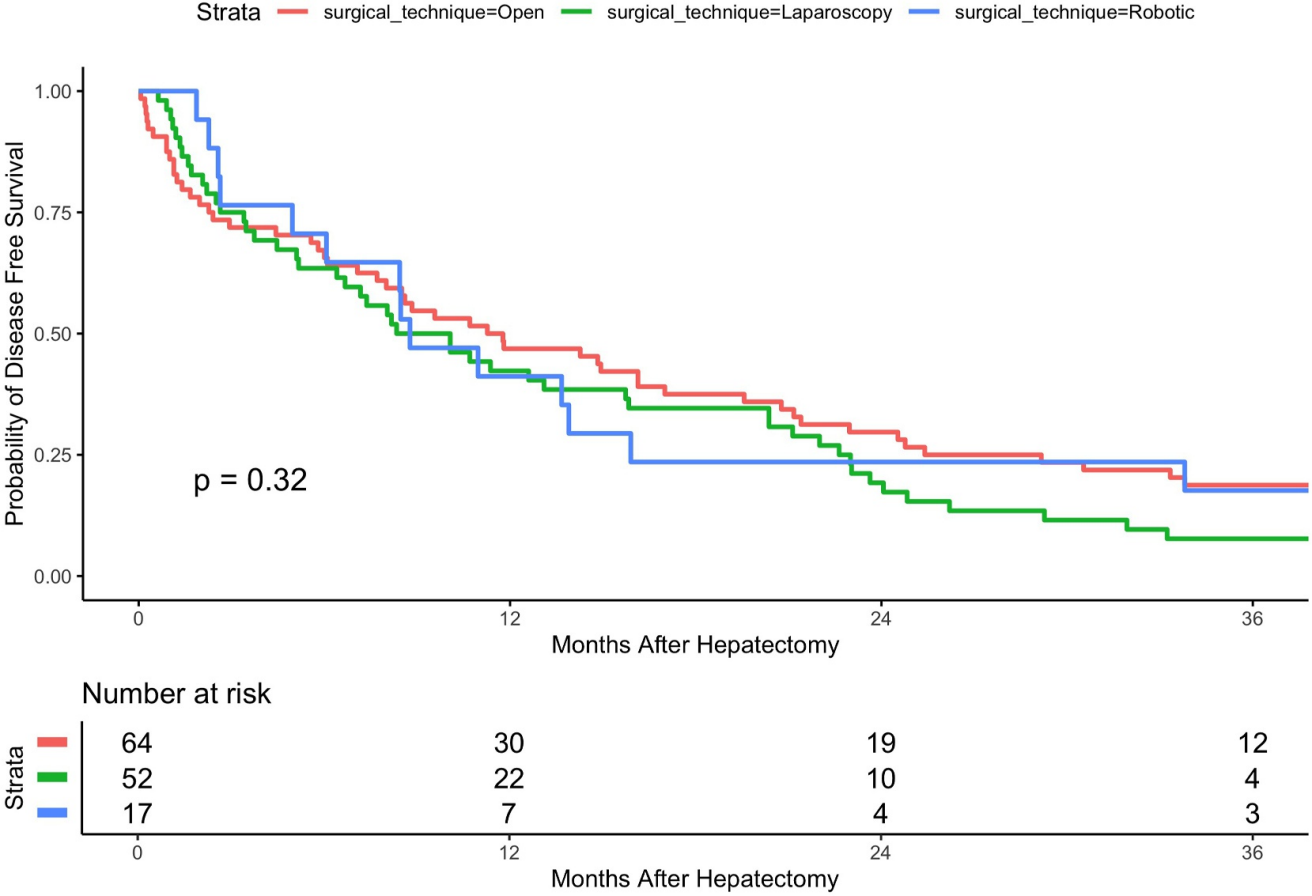
Kaplan‐Meier disease‐free survival estimation at 1 and 3 years, according to the surgical approach.

Kaplan–Meier overall survival estimates at 1 and 3 years were 79% and 30% for OLR; 95% and 89% for LLR; and 90% and 81% for RLR, respectively (*p* < 0.001) (Figure 4). Disease‐free survival estimates showed no significant differences across the groups at 1 and 3 years (Figure 5).

#### 3.3.3 Sensitivity Analysis

Leave‐One‐Center‐Out (LOCO) analysis showed that, compared with OLR, LLR were consistently associated with a lower hazard of death across centers (HR ∼0.25), with 95% confidence intervals not crossing 1. RLR also were associated to a reduced hazard (HR ∼0.40), although confidence intervals were wider and approached 1 in some centers, indicating greater variability (Figure S1). Overall, the LOCO analysis suggests that the observed survival benefit of minimally invasive approaches is not driven by a single center, but that the magnitude of effect is somewhat center‐dependent for RLR.

### 3.4 Predictors of Survival Outcomes

In univariable Cox regression analysis, several factors were significantly associated with overall mortality whereas benchmark failure (absence of lymphadenectomy and/or R1 resection) was marginally associated with higher mortality (HR = 1.55, 95% CI 1.01–2.37, *p* = 0.045). The presence of multiple liver lesions (HR = 2.68, 95% CI 1.70–4.23, *p* < 0.001), tumor size > 5 cm (HR = 1.80, 95% CI 1.15–2.83, *p* = 0.011), cirrhosis (HR = 0.26, 95% CI 0.09–0.71, *p* = 0.009), and high surgical difficulty (IMM 3) (HR = 2.42, 95% CI 1.11–5.29, *p* = 0.027) were associated with increased risk of death. In contrast, laparoscopic (HR = 0.13, 95% CI 0.05–0.33, *p* < 0.001) and robotic approaches (HR = 0.37, 95% CI 0.15–0.91, *p* = 0.030) were associated with a significantly lower mortality risk compared to open surgery.

In the multivariable analysis, only the presence of multiple lesions (HR = 2.18, 95% CI 1.30–3.65, *p* = 0.003) and the laparoscopic approach (HR = 0.15, 95% CI 0.05–0.46, *p* < 0.001) remained independently associated with overall mortality. Robotic surgery was also associated with a decreased risk of death (HR = 0.37, 95% CI 0.13–1.02), although this did not reach statistical significance (*p* = 0.054). Other variables, including cirrhosis, tumor size, ASA classification, surgical difficulty, and benchmark status, were not independently associated with overall survival in the multivariable model (Table 2).

**TABLE 2:** Univariable and multivariable survival analysis to identify predictors of death.

Characteristic	Univariable			Multivariable		
	HR	95% CI	*p*‐value	HR	95% CI	*p*‐value
Sex						
*Female (ref.)*	—	—		—	—	
Male	1.26	0.82–1.93	0.3			
Age class						
*< 50 years (ref.)*	—	—		—	—	
50–70 years	0.87	0.41–1.83	0.7			
> 70 years	1.49	0.71–3.17	0.3			
Surgical approach						
*Open (ref.)*	—	—		—	—	
Laparoscopy	0.13	0.05–0.33	< 0.001	0.15	0.05–0.46	< 0.001
Robotic	0.37	0.15–0.91	0.03	0.37	0.13–1.02	0.054
BMI > 30						
*No (ref.)*	—	—		—	—	
Yes	0.65	0.32–1.33	0.2			
Metabolic syndrome						
*No (ref.)*	—	—				
Yes	0.55	0.28–1.06	0.075			
Cirrhosis						
*No (ref.)*	—	—		—	—	
Yes	0.26	0.09–0.71	0.009	0.91	0.27–3.08	0.9
ASA class						
*I (ref.)*	—	—		—	—	
II	1.43	0.70–2.92	0.3			
III	1.23	0.58–2.61	0.6			
IV	4.46	0.56–35.5	0.2			
Number of lesions						
*Single (ref.)*	—	—		—	—	
Multiple	2.68	1.70–4.23	< 0.001	2.18	1.30–3.65	0.003
Tumor size						
*< 5 cm (ref.)*	—	—		—	—	
> 5 cm	1.8	1.15–2.83	0.011	0.9	0.53–1.52	0.7
IMM surgical difficulty						
*I—Minor (ref.)*	—	—		—	—	
II—Intermediate	1.31	0.53–3.24	0.6	1.33	0.52–3.42	0.5
III—Major	2.42	1.11–5.29	0.027	1.2	0.53–2.72	0.7
Oncological benchmark (R0 + LND)						
*Not achieved (ref.)*	—	—		—	—	
Achieved	1.55	1.01–2.37	0.045	1.14	0.72–1.82	0.6

*Note:* Cox proportional hazards regression. Reference categories are shown in italics. Green shading indicates *p* < 0.05. Dashes (—) indicate the reference category.

Abbreviations: ASA, American Society of Anesthesiologists; BMI, Body Mass Index; CI, Confidence Interval; HR, Hazard Ratio; IMM, Institut Mutualiste Montsouris Difficulty Classification; LND, Lymphadenectomy.

In the secondary analysis separating the benchmark components, R1 resection and lymphadenectomy (LND) were *associated* with poorer survival in univariable analysis, but only LND remained significant after adjustment (HR 4.72, 95% CI 1.20–18.5; *p* = 0.026). Minimally invasive approaches suggested improved survival univariably, though these associations were attenuated in multivariable models. Multiple lesions remained an independent predictor of worse outcomes, whereas tumor size > 5 cm and cirrhosis were not associated with survival after adjustment (Table S1).

## 4 Discussion

This study primarily investigates the impact of benchmark attainment on long‐term outcomes after ICC resection. The three surgical approaches—open (OLR), laparoscopic (LLR), and robotic (RLR)—are reported to reflect real‐world practice within this multicenter cohort from 22 high‐volume centers. The composite oncological benchmark—R0 resection combined with lymphadenectomy (LND)—was achieved in 38.8% of patients, with higher rates in the OLR (51.1%) and RLR (44.4%) groups compared with LLR (14.5%) (*p* < 0.001). These findings suggest that among minimally invasive approaches, RLR may more closely approximate the benchmark performance of OLR [15] compared to LLR, as suggested by available evidence [12, 14]. However, higher benchmark adherence did not translate into improved survival, highlighting a potential disconnect between surrogate surgical quality metrics and long‐term oncologic outcomes.

When examined separately, the R1 resection rate remained high across the cohort (39.6%), exceeding the recommended benchmark [6] threshold of 27.5%, particularly in the OLR (47.4%) and RLR (33.3%) groups. Only LLR approached the benchmark threshold (27.6%, *p* = 0.014), though margin width differences did not persist after adjusting for surgical difficulty, suggesting case complexity alone does not account for margin status.

Lymphadenectomy was performed in 69.6% of patients overall, exceeding historical rates reported in ICC benchmark studies (only 42.8% in ICC resections [6, 10]). It is important to remind that the primary established value of LND lies in staging accuracy and prognostication to guide adjuvant therapy decisions, while its direct therapeutic impact remains unproven [22, 23]. Recent large‐scale data [24] have reaffirmed this central role of adequate lymph node evaluation (LNE), with an analysis of over 6500 patients from the National Cancer Database showing that adequate lymphadenectomy (≥ 6 nodes) significantly improves risk stratification. Notably, the definition of an adequate LND has evolved over time, with the minimum required number of nodes increasing in successive AJCC guidelines [6, 8]. This historical variability explains our choice to use a pragmatic “LND performed: yes/no” criterion rather than a specific node count threshold for this multicenter study spanning several years. In the present study, LND rates varied widely by approach: 97.8% in OLR, 59.3% in RLR, and only 22.4% in LLR (*p* < 0.001). Although the number of retrieved nodes was similar, the markedly lower LND rate in LLR raises concerns regarding understaging and oncological completeness. These concerns are consistent with previous nationwide French studies [12, 13], which showed that LLR was associated with lower rates of LND and adjusted textbook outcome, despite perioperative benefits such as reduced transfusion rates and shorter hospital stays. These findings highlight the need for standardized nodal dissection protocols in minimally invasive surgery for ICC, even as the therapeutic benefit of LND continues to be evaluated. Interestingly, despite lower benchmark adherence, survival outcomes were more favorable in the MIS group. Both LLR and RLR were associated with improved overall survival at 1 and 3 years compared to OLR, with LLR remaining an independent protective factor in multivariable analysis (HR 0.15, 95% CI 0.05–0.46; *p* < 0.001). RLR showed a similar trend (HR 0.37, 95% CI 0.13–1.02; *p* = 0.054). These results suggest that survival benefits observed with MIS may stem less from oncological radicality than from reduced perioperative morbidity or selection of patients with more favorable disease biology.

Notably, 90‐day postoperative mortality in the cohort was 5.0% (12/240), closely aligned with the proposed international benchmark of ≤ 4.8% for ICC [6], without significant differences between surgical approaches (Fisher's exact test, *p* = 0.219). This figure confirms the overall perioperative safety of this real‐world multicenter cohort, despite its inclusion of high‐complexity procedures (56.7% IMM class III) and high‐risk patients across more than 40 centers over 14 years. Mortality rates were numerically lower in the LLR group (2.6%) and the RLR group (0%) compared with OLR (7.3%), consistent with the well‐established selection of less complex, lower‐risk cases for minimally invasive approaches. Causes of postoperative death were predominantly related to severe sepsis or major cardiopulmonary events; only two deaths were attributable to early tumor recurrence within the first 90 days, confirming that perioperative mortality in this cohort reflects surgical complexity and patient comorbidities rather than disease aggressiveness. Taken together, these findings validate the perioperative quality of care across participating centers and support the robustness of the long‐term survival analyses.

In the multivariable model, tumor multiplicity remained the only independent oncological predictor of mortality, reinforcing the prognostic impact of tumor burden. Tumor size > 5 cm, cirrhosis, and surgical difficulty lost significance after adjustment, suggesting these factors may be confounded by selection bias or collinearity.

Altogether, these findings reinforce that long‐term prognosis in ICC is primarily influenced by tumor biology and morphological subclasses [25] rather than surgical technique. ICC is a heterogeneous disease characterized by diverse molecular subtypes, systemic inflammatory responses [26]. In this context, the choice of surgical technique plays a secondary role compared with the underlying biological aggressiveness of the disease and the preoperative risk profile of the patient. Integrating molecular and biological markers—such as CA 19‐9, albumin, neutrophil‐to‐lymphocyte ratio [19, 27], and tumor burden scores [28]—into preoperative assessment may improve patient stratification and help identify those most likely to benefit from surgery. Ultimately, the selection of patients for a specific surgical approach should be part of a comprehensive, biology‐driven treatment plan, ensuring that operative decisions are aligned with the tumor's individual behavior and the patient's overall oncologic outlook. To conclude, the observed disconnection between technical benchmarks and survival, are further contextualized by growing evidence that a patient's socioeconomic environment and underlying health disparities are powerful determinants of oncologic outcomes. A recent nationwide study from Sweden's universal healthcare system reported that low socioeconomic status was independently associated with a higher incidence of iCCA, a more advanced stage at diagnosis, lower odds of receiving systemic therapy, and a significantly increased mortality risk, even among treated patients [29]. This underscores that survival after surgery is not solely a function of surgical technique or margin status, but is deeply embedded in a complex web of biology, patient‐specific factors, and social determinants of health that influence both disease aggressiveness and resilience to treatment [30].

### 4.1 Limitations

Several limitations of this study must be acknowledged. The multicenter design—spanning more than 40 centers over 14 years—reflects the real‐world context of iCCA, a rare disease, and naturally entails heterogeneous caseloads, expertise levels, and follow‐up practices. While this variability enhances external validity and provides a representative national snapshot, it may also have influenced perioperative and oncological outcomes, particularly during the early adoption phases of laparoscopic and robotic liver surgery. Because of inter‐center heterogeneity and the learning curves inherent to minimally invasive techniques, direct comparisons between open, laparoscopic, and robotic resections remain vulnerable to unmeasured confounding and selection bias, and should therefore be interpreted as hypothesis‐generating. As this is an observational and retrospective study, we were limited in our ability to use randomization or case‐control matching between groups, due in part to the small number of patients undergoing RLR. We would have liked to stratify based on surgical indication to reduce the risk of selection bias. We used multivariate Cox regression to reduce treatment allocation bias; a propensity score approach could also have been considered. The low number of RLR patients (*n* = 27) also raises the possibility of a type II error for comparisons involving the robotic group, which may be underpowered to detect meaningful survival differences. However, the LOCO Cox sensitivity analysis—systematically assessing the influence of each covariate—confirmed the stability of the survival model, supporting the robustness of our primary finding that benchmark attainment was not significantly associated with long‐term outcomes. A second key limitation is the substantial baseline imbalance between the three surgical groups, resulting from non‐randomized allocation based on tumor characteristics and patient risk profiles. These differences limit any causal interpretation of survival outcomes across surgical approaches. The apparent survival advantage observed with minimally invasive techniques is thus more likely driven by selection bias, underscoring the predominant influence of patient and tumor factors over the choice of surgical approach in determining long‐term outcomes.

## 5 Conclusion

In this multicenter cohort of patients undergoing liver resection for intrahepatic cholangiocarcinoma, minimally invasive approaches—particularly LLR—were associated with improved long‐term survival in adjusted analyses, despite lower rates of conventional oncologic benchmark attainment. RLR more closely replicated the benchmark performance of OLR, although no independent survival benefit was observed.

These findings suggest a possible mismatch between traditional surgical surrogate markers and oncological outcomes, raising the hypothesis that prognosis may be driven more by underlying tumor biology and patient selection than by the technical approach itself. Further standardization of lymphadenectomy practices and greater integration of biological and molecular factors into surgical decision‐making may help refine patient selection and improve outcomes in ICC.

## Author Contributions

**Delphine Gastineau:** conceptualization, investigation, formal analysis. **Claire Goumard:** investigation. **Alexandre Doussot:** data curation. **Marc‐Antoine Allard:** data curation. **Raphael Venezia:** data curation. **Serena Langella:** data curation. **Guillaume Millet:** data curation. **Ruth Bustamante:** data curation. **Christian Hobeika:** data curation. **Rami Rhaiem:** data curation. **Stylianos Tzedakis:** data curation. **Heithem Jeddou:** data curation. **Bastien Le Floch:** data curation. **Alain Valverde:** data curation. **Nicolas Peru:** data curation. **Patrick Pessaux:** data curation. **Fabio Giannone:** data curation. **Jean Yves Mabrut:** data curation. **Kayvan Mohkam:** data curation. **Antonio Sa Cunha:** data curation. **Chady Salloum:** data curation. **Pierre Yves Blanc:** data curation. **Bertrand Le Roy:** data curation. **Takayuki Kawai:** data curation. **Olivier Farges:** data curation. **Stefan Hofmeyr:** data curation. **David Fuks:** data curation. **Stéphanie Truant:** data curation. **Daniel Cherqui:** data curation. **Alessandro Ferrero:** data curation. **Emmanuel Boleslawski:** data curation. **Jean‐Marc Regimbeau:** data curation. **Eric Vibert:** data curation. **Daniele Sommacale:** validation. **Olivier Soubrane:** resources, data curation. **Olivier Scatton:** resources, data curation. **Alexis Laurent:** resources, data curation. **Raffaele Brustia:** writing – original draft, writing – review and editing, supervision.

## Funding

The authors have nothing to report.

## Conflicts of Interest

The authors declare no conflicts of interest.

## Supporting information


**Figure S1:** Forest plot summarizing hazard ratios with 95% confidence intervals from the LOCO Cox regression analysis. Each line represents the effect of removing one covariate from the full model, highlighting its individual contribution to overall survival.

**Table S1:** Univariable and multivariable survival analysis to identify predictors of death including each element of the composite outcome “benchmark”.

## Data Availability

The data that support the findings of this study are available on request from the corresponding author. The data are not publicly available due to privacy or ethical restrictions.
