# Supplementary material for: Does Surgical Approach Influence Benchmark Attainment in Intrahepatic Cholangiocarcinoma?
Source: World J Surg. 2026 Jul 24;50(9):2714–25. doi: 10.1002/wjs.70491 (PMC13576594; doi:10.1002/wjs.70491)
Supplement: Supplementary file 1 [file WJS-50-2714-s002.docx]

Supplementary table 1

| UNIVARIABLE | | | | |  | |  | |  | | MULTIVARIABLE | | | | | |  |  |
| --- | --- | --- | --- | --- | --- | --- | --- | --- | --- | --- | --- | --- | --- | --- | --- | --- | --- | --- |
| Characteristic | | HR*^1^* | | | 95% CI*^1^* | | p-value | |  | | HR*^1^* | | 95% CI*^1^* | | p-value | |  |  |
| Sex | | | | |  | |  | |  | |  | |  | |  | |  |  |
| F |  | | — | | | — | |  | |  | |  | |  | |  | |  |
| M | 1,26 | | | | 0,82 – 1,93 | | 0,3 | |  | |  | |  | |  | |  |  |
| Surgical approach | | | | |  | |  | |  | |  | |  | |  | |  |  |
| Open | | — | | | — | |  | |  | | — | | — | |  | |  |  |
| Laparoscopy | | 0,13 | | | 0,05 – 0,33 | | <0,001 | |  | | 0.32 | | 0.09, 1.22 | | 0.10 | |  |  |
| Robotic | | 0,37 | | | 0,15 – 0,91 | | 0,03 | |  | | 0.35 | | 0.12, 1.01 | | 0.052 | |  |  |
| BMI > 30 | |  | | |  | |  | |  | |  | |  | |  | |  |  |
| 0 | | — | | | — | |  | |  | |  | |  | |  | |  |  |
| 1 | | 0,65 | | | 0,32 – 1,33 | | 0,2 | |  | |  | |  | |  | |  | |
| Metabolic syndrome | |  | | |  | |  | |  | |  | |  | |  | |  | |
| 0 | | — | | | — | |  | |  | |  | |  | |  | |  | |
| 1 | | 0,55 | | | 0,28 – 1,06 | | 0,075 | |  | | 0.43 | | 0.19, 0.97 | | **0.042** | |  | |
| Cirrhosis | |  | | |  | |  | |  | |  | |  | |  | |  | |
| 0 | | — | | | — | |  | |  | | — | | — | |  | |  |  |
| 1 | | 0,26 | | | 0,09 – 0,71 | | 0,009 | |  | | 1.18 | | 0.34, 4.09 | | 0.8 | |  |  |
| ASA | |  | | |  | |  | |  | |  | |  | |  | |  |  |
| 1 | | — | | | — | |  | |  | |  | |  | |  | |  |  |
| 2 | | 1,43 | | | 0,70 – 2,92 | | 0,3 | |  | |  | |  | |  | |  |  |
| 3 | | 1,23 | | | 0,58 – 2,61 | | 0,6 | |  | |  | |  | |  | |  |  |
| 4 | | 4,46 | | | 0,56 – 35,5 | | 0,2 | |  | |  | |  | |  | |  |  |
| Number of lesions | | | | | | |  | |  | |  | |  | |  | |  |  |
| Single | | — | | | — | |  | |  | | — | | — | |  | |  |  |
| Multiple | | 2,68 | | | 1,70 – 4,23 | | <0,001 | |  | | 2.44 | | 1.40, 4.25 | | **0.002** | |  |  |
| Tumor>5cm | |  | | |  | |  | |  | |  | |  | |  | |  |  |
| <5cm | | — | | | — | |  | |  | | — | | — | |  | |  |  |
| >5cm | | 1,8 | | | 1,15 – 2,83 | | 0,011 | |  | | 0.83 | | 0.48, 1.41 | | 0.5 | |  |  |
| IMM | |  | | |  | |  | |  | |  | |  | |  | |  |  |
| 1 | | — | | | — | |  | |  | | — | | — | |  | |  |  |
| 2 | | 1,31 | | | 0,53 – 3,24 | | 0,6 | |  | | 1.45 | | 0.56, 3.73 | | 0.4 | |  |  |
| 3 | | 2,42 | | | 1,11 – 5,29 | | 0,027 | |  | | 1.14 | | 0.51, 2.58 | | 0.7 | |  |  |
| R status | | | | |  | |  | |  | |  | |  | |  | |  |  |
| 0 | | — | | | — | |  | |  | | — | | — | |  | |  |  |
| 1 | | 1.68 | | | 1.09, 2.57 | | 0.018 | |  | | 1.18 | | 0.74, 1.88 | | 0.5 | |  |  |
| Lympnadenectomy (LND) | | | |  |  | |  | |  | |  | |  | |  | |  |  |
| 0 | | — | | | — | |  | |  | | — | | — | |  | |  |  |
| 1 | | 9.63 | | | 3.53, 26.3 | | <0.001 | |  | | 4.72 | | 1.20, 18.5 | | 0.026 | |  |  |
| *^1^* HR : Hazard Ratio, CI : Confidence Interval, ASA: American Society of Anesthesiologists; BMI: Body Mass Index; IMM : *Institut Mutualiste Montsouris* Difficulty classification | | | | | | | | | | | | | | | | |  |  |
